# Feature Integration and Task Switching: Diminished Switch Costs after Controlling for Stimulus, Response, and Cue Repetitions

**DOI:** 10.1371/journal.pone.0151188

**Published:** 2016-03-10

**Authors:** James R. Schmidt, Baptist Liefooghe

**Affiliations:** Department of Experimental Clinical and Health Psychology, Ghent University, Ghent, Belgium; Vrije Universiteit Brussel, BELGIUM

## Abstract

This report presents data from two versions of the task switching procedure in which the separate influence of stimulus repetitions, response key repetitions, conceptual response repetitions, cue repetitions, task repetitions, and congruency are considered. Experiment 1 used a simple alternating runs procedure with parity judgments of digits and consonant/vowel decisions of letters as the two tasks. Results revealed sizable effects of stimulus and response repetitions, and controlling for these effects reduced the switch cost. Experiment 2 was a cued version of the task switch paradigm with parity and magnitude judgments of digits as the two tasks. Results again revealed large effects of stimulus and response repetitions, in addition to cue repetition effects. Controlling for these effects again reduced the switch cost. Congruency did not interact with our novel “unbiased” measure of switch costs. We discuss how the task switch paradigm might be thought of as a more complex version of the feature integration paradigm and propose an episodic learning account of the effect. We further consider to what extent appeals to higher-order control processes might be unnecessary and propose that controls for feature integration biases should be standard practice in task switching experiments.

## Introduction

Mental flexibility is a hallmark of executive functioning [1–3]. That is, our ability to shift our thinking from one concept to another is deemed a crucial aspect of higher-order cognitive functioning. In attempting to measure such cognitive control processes, however, many lower level learning or memory biases are often a concern. For instance, it is known that the repetition of stimulus or response features can have a considerable effect on performance [4, 5]. These repetition effects can often represent confounds in “cognitive control” tasks (e.g., proportion congruent and congruency sequence tasks), because they are often not equally distributed among the various conditions in an experiment (e.g., [6]). For instance, it has been argued that one supposed cognitive control phenomenon, the congruency sequence effect, might be explained by unintentional confounding of feature repetitions in the task [7–9] (for a review, see [10]).

**Fig 1:**
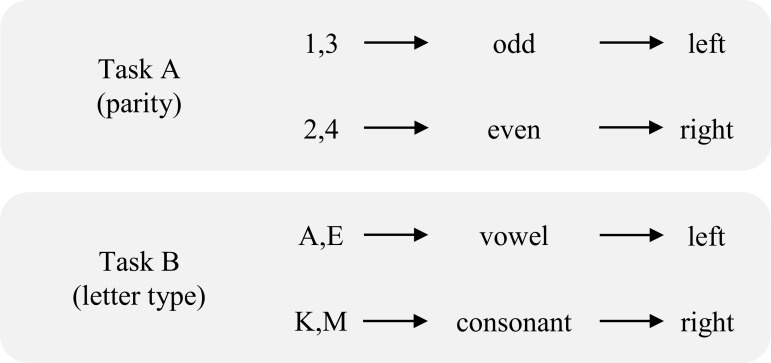
Example representation of the tasks used in Experiment 1.

Other paradigms might suffer from similar problems. In particular for the present report, we ask whether feature repetitions also contribute, and perhaps even fully explain, the switch cost? The *switch cost* is the observation that responses are considerably slower when the task on the previous trial (e.g., parity judgment of digits) is different than the task on the current trial (e.g., consonant/vowel decisions of letters), relative to when the task repeats [11] (for reviews, see [12–14]). An example of two tasks is presented in Fig 1. Quite frequently, accounts of the switch cost center around the concept of *task sets*. Although there are some ambiguities in what constitutes a “task set” [15], we here borrow a definition from Vandierendonck and colleagues [14]: “a collection of control settings or task parameters that program the system to perform processes such as stimulus identification, response selection, and response execution” (p. 601). Accounts of the switch cost are typically interpreted in terms of cognitive control processes engaged during transitions between task sets. For instance, the switch cost is often interpreted in terms of a task reconfiguration cost on a task switch (e.g., [16, 17]). That is, it is argued that the task set needs to be changed when the task on the previous trial is different than the task on the current trial. Another view proposes that the switch cost is related to proactive interference between the currently- and previously-active task sets (e.g., [18]). Such accounts thus propose that the switch cost, or at least much of it, reflects higher-order control of task sets.

**Fig 2:**
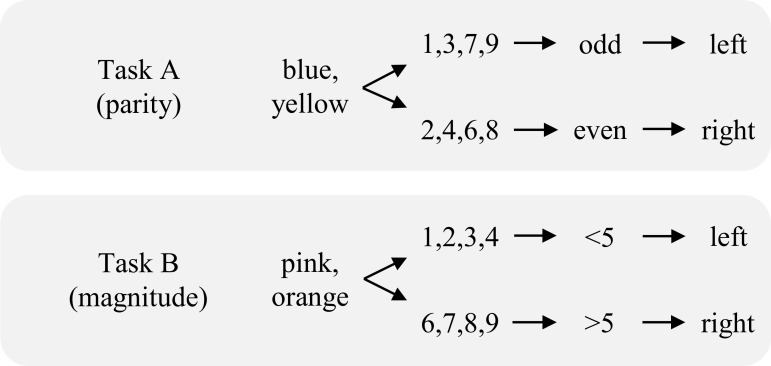
Example representation of the tasks used in Experiment 2.

However, there are alternative accounts which propose an entirely different mechanism. Rather than appealing to higher-order control processes, these accounts suggest that lower-level cue, stimulus, or response transitions explain the switch cost [19–23]. For instance, Logan and Bundesen [19, 20] argued that the cued version of the task switch paradigm (i.e., in which a pre-presented cue indicates which of the two tasks to perform on the upcoming trial) might be fully, or at least primarily, driven by cue repetition benefits. Example stimuli for such a design are presented in Fig 2. More specifically, if there are two cues that indicate Task A and two other cues that indicate Task B, then it is possible for the cue to repeat (or not) if the task repeats. It is impossible for the cue to repeat if the task alternates. Logan and Bundesen demonstrated very large cue repetition benefits. Furthermore, controlling for these cue repetition benefits accounts for the majority of the “task” switch cost. Though the entire effect does not appear to be explained by such repetitions [24, 25], most of it is. This would suggest that much of the switch cost has little to do with a change in the task itself, contrary to the reconfiguration cost and proactive interference notions.

Cue repetition effects are only one of many factors that can bias switch costs. As we will propose in the current work, the “true” switch costs that remain after controlling for cue repetitions are further complicated by other confounds. Of particular interest for the current work, a large part of this switch cost might actually be due to *feature integration biases*, because the types of features that can or cannot repeat is different on task alternations and task repetitions. Performance is very fast when making the same response to the same stimulus as in the previous trial, termed a *complete repetition*, and when making a different response to a different stimulus, termed a *complete alternation* [5]. In contrast, performance is hindered when making (a) a *different* response to the *same* stimulus or (b) the *same* response to a *different* stimulus, both termed a *partial repetition*. Note that while the terms “repetition” and “switch” are typically used in the task switching literature, the terms “repetition” and “alternation” are typically used in feature integration work. To avoid potential confusion, we use the term “alternation” consistently throughout the paper.

As a concrete example of how feature integration biases impact the switch cost, consider the version of the task switching paradigm in which participants respond to digits with a parity (odd/even) decision in one task and to letters with a consonant/vowel decision in the other task (e.g., [17]; see Fig 1). With a task repetition, it is possible for an exact *stimulus repetition* of the attended stimulus. For instance, after responding to the letter “M” another “M” might be presented as the imperative stimulus (M→M). Because participants make the same response to the same stimulus (i.e., complete repetition), performance will benefit. Such a stimulus repetition is *not* possible when the task changes, because the target stimulus alternates from a letter to a digit, or vice versa. Thus, faster performance in the task repetition condition might, in part, be explained by these stimulus repetitions. Although stimulus repetitions are sometimes disallowed in the procedure or trimmed after the fact [26–28], this is not always the case.

Of course, other versions of the task switch paradigm do allow for stimulus repetitions on both task alternations and repetitions. For instance, consider a cued version of the paradigm in which participants always respond to digits, but with either a parity or magnitude judgment, depending on the cue (see Fig 2). The two tasks do not have two different sets of stimuli. However, a repeated stimulus *always* entails the same response on a task repetition (complete repetition), but sometimes requires a different response on a task alternation (partial repetition).

Some evidence indicates that such feature integration biases probably do impact the switch cost. For instance, Goschke [29] found differences in performance between complete repetition/alternation and partial repetition trials, which contributed (but did not fully explain) the switch cost (see also, [30, 31]). Although results such as this seem to indicate that feature integration between stimuli and responses plays an important role in switching experiments, most research so far has neglected to control for feature integration biases. As we will argue, the presence of these biases results in a systematic overestimation of the magnitude of switch costs. As a result, the switch cost might have more to do with low-level repetition biases, and less to do with higher-order control.

Yet another potential contributor to the switch cost is a *physical response repetition* bias. That is, the response a participant has to make (e.g., left or right key press) can repeat (e.g., left→left) or alternate (e.g., left→right). If physical response repetitions are faster than alternations [32], then this might matter for the switch cost. Physical response repetitions might initially seem to be less problematic, as they should be equally distributed among task repetitions and alternations. However, this is complicated by the *interactions* response repetitions can have with other types of repetitions. We already discussed how making a different response to the same stimulus can impair performance. We discuss a further complication in the following.

Yet another type of potential “bias” in the switch cost are *conceptual response repetitions* [33]. Different than the actual physical response, the conceptual response can repeat (e.g., odd→odd) or alternate (e.g., odd→even or odd→consonant). A conceptual response repetition, which benefits performance [28, 33–35], can only occur with a task repetition. In contrast, on a task alternation the most that can repeat is the physical key press response, not the actual conceptual response. For instance, if even digits and consonants are responded to with the same response key (e.g., the right key), then the physical response can repeat on a task alternation (right→right) but the conceptual response cannot (even→consonant). Thus, conceptual response repetitions might also explain part of the switch cost.

Not only are conceptual response repetitions only possible on a task repetition, thus producing a response repetition *benefit*, but it is also the case that a repeated physical response on a task alternation entails making a different conceptual response with the same physical response. This “partial repetition” might thus produce an impairment in performance on response repetitions during a task alternation. Indeed, several authors have studied response repetition benefits and costs within the context of task switching experiments [17, 26–28, 33, 36, 37]. Typically, there is a response repetition *benefit* for task repetitions, but a response repetition *cost* for task alternations. Furthermore, Altmann [36] (see also, [21, 23]) observed that the response repetition benefit is particularly large on task repetitions with a cue repetition. Although Altmann was not primarily concerned with the switch cost itself, these results lend credence to our suggestion that the bindings of conceptual and physical responses impacts the switch cost.

Similarly, Meiran [33] provides a mathematical model in which the connection between a physical and conceptual response are strengthened after being linked together (e.g., *odd* with the left key) and the connection between a physical response and the conceptual response that was *not* made on that trial (e.g., *less than five* and the left key) are weakened. This means that it will be easier to make the same conceptual response (e.g., odd) with the same key (left) on the next trial (complete repetition), but harder to make a different conceptual response (less than five) with the same key (left; partial repetition). Although this past work was primarily concerned with response repetition effects directly, it is also important to note the confound that these biases represent for the switch cost. Easy complete repetitions (i.e., same concept, same key) are only possible on a task repetition. Hard partial repetitions (i.e., different concept, same key) are only possible on a task alternation. Thus, any “pure” task switch cost is confounded with conceptual-physical response binding effects.

As we will argue, binding of differing stimulus, response, and cue features into memory can lead to retrieval biases on subsequent trials that produce a switch cost. These biases alone might provide a sufficient (or perhaps nearly sufficient) account of the switch cost. We note that this notion is related to some previous work assessing the role of stimulus bindings to tasks [38–40]. Specifically, it has been proposed that stimuli previously presented with a different task are linked in memory to the task it was encountered with. This leads to feature integration costs when the same stimulus is presented with a new task, which contributes to the switch cost. We will expand on this idea in the Discussion section, but note that it is slightly different than the account we suggest, still proposing a role for higher-order control functions (i.e., the linking of task sets to stimuli).

We suggest that feature integration processes systematically bias the switch cost, thereby explaining much of it. Novel to the current paper, we present an integrative approach, in which we simultaneously consider various types of feature integration biases concurrently. As we will demonstrate, this is important as the magnitude of the impact of feature integration biases on the switch cost can only be properly appreciated by illustrating the net impact of all biases at once (i.e., as opposed to controlling for one sort of bias, but neglecting others). That said, our account does share some similarities with the account of response repetition benefits and costs presented by Altmann [36], with two differences. First, we present our episodic model as an account of the switch cost, not merely response repetition effects. Second, we propose that the task itself may not be particularly relevant in this episodic binding process. We feel that our account is also comparable to the work of Logan and colleagues on cue repetition effects (e.g., [20]), except that we expand our analysis beyond just cue repetition effects. Specifically, we suggest that considering bindings involving cues, stimuli, and responses, and argue that such bindings might be sufficient to account for much of the switch cost, without needing to appeal to the notion of retrieval of prior tasks. Also novel to the present report, we argue that controls for feature integration biases should become standard practice in task switching research, as is the case in other literatures (for a discussion, see [6]).

## Experiment 1

In order to test for these potentially confounding factors, we ran a simple alternating runs procedure [17] in Experiment 1. That is, the experiment alternated between pairs of trials from two tasks. Participants were presented with a single stimulus, either a letter or a digit. One task was letter type identification (vowel vs. consonant) and the other task was digit parity identification (odd vs. even; e.g., similar to [17], but with univalent stimuli). We were then able to generate five types of trials. For condition names, we use a notation to help keep track of the task, stimulus, and response repetitions in each trial type. Specifically, each condition is represented by the prefix “*rep*” or “*alt*” for task repetitions and alternations, respectively. This is then followed by two serial letters, such as “*AR*,” with “*R*” for repetition and “*A*” for alternation. The first letter in the pair represents the repetition or alternation of the stimulus. The second letter refers to the response. Thus, for instance, “*alt-AR*” means that the task alternates, the stimulus alternates, and the response repeats. Note that conceptual response repetitions are not represented in this notation, but physical response repetitions are always a conceptual response repetition on a task repetition, and always a conceptual response alternation on a task alternation.

The five conditions that our manipulation produces are presented in Table 1. Three of the five conditions that our manipulation produced were task repetitions (e.g., parity judgment followed by parity judgment). In *rep-RR* trials, both the stimulus and the response repeat (e.g., 2→2). In *rep-AR* trials, the response repeats but the stimulus changes (e.g., 2→4). The difference between these two conditions thus indexes a stimulus repetition benefit. In *rep-AA* trials, both the stimulus and response change (e.g., 2→3). The difference between *rep-AR* and *rep-AA* is thus the response repetition effect (both the conceptual and physical response).

**Table 1:** Five trial types in Experiment 1.

	Repetition Type			
Condition	Task	Stimulus	Conceptual Response	Physical Response
rep-RR	✓	✓	✓	✓
rep-AR	✓	✗	✓	✓
rep-AA	✓	✗	✗	✗
alt-AR	✗	✗	✗	✓
alt-AA	✗	✗	✗	✗

The remaining two conditions were task alternations. In *alt-AR* trials, the physical key response repeats, but the stimulus changes (e.g., 2→M, assuming even digits and consonants are mapped to the same key). Finally, in *alt-AA* trials, both the stimulus and response change (e.g., 2→A). The only difference between *alt-AR* and *alt-AA* is the repetition or alternation of the physical key response. Because the physical response is linked to a new conceptual response, we should expect a response repetition *cost*. Finally, the only difference between *alt-AA* and *rep-AA* is the repetition of the task, thus allowing a test of the switch cost independent of a stimulus or response repetition.

Altogether then, we have independent tests of task repetitions (*alt-AA*–*rep-AA*), stimulus repetitions (*rep-AR*–*rep-RR*), response repetition benefits when the conceptual response repeats (*rep-AA*–*rep-AR*), and response repetition costs when the conceptual response alternates (*alt-AA*–*alt-AR*). Conceptual response repetitions cannot necessarily be assessed independent of physical response repetitions (see Table 1), but the binding of conceptual and physical responses can be.

Note that while switching between two stimulus sets is less common than using one stimulus set with two tasks (and tends to produce smaller switch costs; e.g., [17, 18]), the current design does allow us to assess stimulus and response repetition effects in a paradigm that is not confounded by congruency effects. When the same stimuli are used for two different tasks, the response to a given stimulus can either be: (a) *congruent*, engendering the same response in both tasks, or (b) *incongruent*, engendering a different response in the two tasks [16, 17, 41]. Congruent stimuli are responded to faster than incongruent stimuli, and thus taking into consideration these additional congruency relations would complicate our analyses. We return to this point later in the paper.

### Method

#### Participants

Thirty-eight Ghent University undergraduates participated in the study in exchange for €5. This study was approved by the Ethics Committee of Ghent University. Participants provided written consent before participating.

#### Apparatus

Stimulus presentation was controlled by E-Prime 2 (Psychology Software Tools, Pittsburgh, PA). Responses were made on an AZERTY keyboard using the “F” and “J” keys with the left and right index fingers, respectively.

#### Design

White 18pt Courier New bold stimuli were presented on a black background. There were eight stimuli in total, consisting of four uppercase letters and four digits. Two of the letter stimuli were consonants (K, M) and two were vowels (A, E). Two of the digit stimuli were odd (1, 3) and two even (2, 4). Half of the participants responded to consonants with the “F” key and vowels with the “J” key, while the other half had the reverse mappings. Orthogonal to this, half of the participants responded to odd digits with the “F” key and even digits with the “J” key, while the other half had the reverse mappings. As previously mentioned, these manipulations allow for five types of trials: *rep-RR*, *rep-AR*, *rep-AA*, *alt-AR*, and *alt-AA*.

#### Procedure

On each trial, participants saw a white fixation “+” for 150ms, followed by a blank screen for 150ms, followed by a single target stimulus (i.e., a letter or a digit) for 2000ms or until a response was made. Following correct responses, the next trial immediately began. Following incorrect responses and trials in which participants failed to respond in 2000ms, “XXX” in white was presented for 500ms before the next trial. There were 300 trials in total, presented in one large block. Trials followed a fixed, repeating sequence of letter, letter, digit, digit, etc. This was mentioned to participants on the instruction screen. Each letter or digit was selected at random with replacement on each trial out of the four possible stimuli.

### Results

Mean correct RT and percentage error data were analyzed. All trials in which participants failed to respond in 2000ms (<1% of trials) and trials for which an error was made on the previous trial were excluded from analyses.

#### Response times

First, we conducted the traditional analysis, ignoring the distinctions between the repetition or alternation of stimuli, conceptual responses, and physical responses. There was a significant benefit for task repetitions (562ms) over task alternations (647ms; effect: 85ms), *t*(37) = 8.403, *SE*_*diff*_ = 10, *p* < .001, $\eta_{p}^{2}=.66$. Thus, we replicated the standard switch cost.

**Fig 3:**
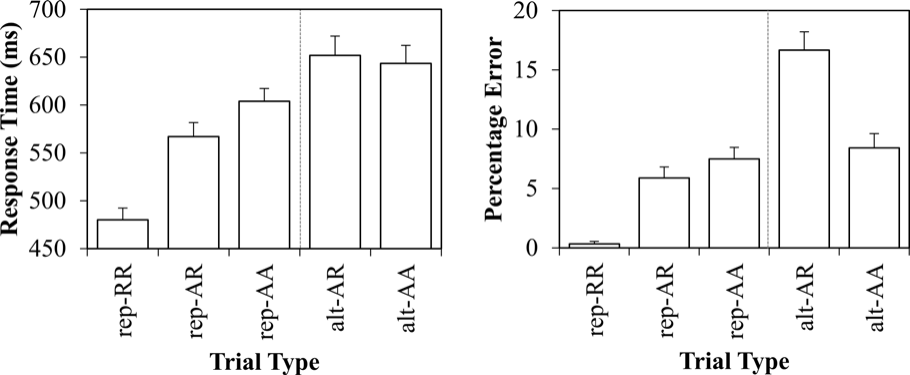
Experiment 1 response times in milliseconds (left) and percentage errors (right) with standard error bars.

Next, an ANOVA was conducted using the within factor of trial type (*rep-RR*, *rep-AR*, *rep-AA*, *alt-AR*, *alt-AA*). The mean RT data are presented in the left panel of Fig 3. This ANOVA was significant, *F*(4,148) = 82.816, *MSE* = 2237, *p* < .001, $\eta_{p}^{2}=.69$. Planned comparisons revealed that *rep-RR* trials (480ms) were significantly faster than *rep-AR* trials (567ms; effect: 87ms), *t*(37) = 9.862, *SE*_*diff*_ = 9, *p* < .001, $\eta_{p}^{2}=.72$, indicating a stimulus repetition benefit. *Rep-AR* trials were significantly faster than *rep-AA* trials (604ms; effect: 37ms), *t*(37) = 4.831, *SE*_*diff*_ = 8, *p* < .001, $\eta_{p}^{2}=.39$, indicating a conceptual-physical response repetition benefit. *Rep-AA* trials were significantly faster than *alt-AA* trials (643ms; effect: 39ms), *t*(37) = 3.980, *SE*_*diff*_ = 10, *p* < .001, $\eta_{p}^{2}=.30$, indicating a task repetition benefit. Note that this 39ms task switch cost is considerably smaller than that with the traditional analysis (85ms). Finally, *alt-AR* trials (652ms) did not significantly differ from *alt-AA* trials (effect: −9ms), *t*(37) = −1.130, *SE*_*diff*_ = 8, *p* = .266, $\eta_{p}^{2}=.03$, indicating no cost for repeating a physical key press if the conceptual response changes. This final finding suggests also that the difference between *rep-AR* and *rep-AA* trials is not merely due to a repetition of the same physical key press, but instead due to the repetition of the same conceptual response.

#### Percentage error

In the traditional analysis, there was a significant benefit for task repetitions (5.3%) over task alternations (11.0%; effect: 5.7%), *t*(37) = 6.867, *SE*_*diff*_ = .8, *p* < .001, $\eta_{p}^{2}=.56$. Thus, we replicated the standard task switch cost in errors.

Next, an ANOVA was conducted using the within factor of trial type (*rep-RR*, *rep-AR*, *rep-AA*, *alt-AR*, *alt-AA*). The percentage error data are presented in the right panel of Fig 3. This ANOVA was significant, *F*(4,148) = 35.861, *MSE* = 24.375, *p* < .001, $\eta_{p}^{2}=.498$. Planned comparisons revealed that *rep-RR* trials (0.3%) produced significantly less errors than *rep-AR* trials (5.9%; effect: 5.6%), *t*(37) = 6.052, *SE*_*diff*_ = .9, *p* < .001, $\eta_{p}^{2}=.50$, indicating a stimulus repetition benefit. *Rep-AR* trials produced marginally less errors than *rep-AA* trials (7.5%; effect: 1.6%), *t*(37) = 1.924, *SE*_*diff*_ = .8, *p* = .062, $\eta_{p}^{2}=.09$, indicating a conceptual and/or physical response repetition benefit. *Rep-AA* trials did not differ from *alt-AA* trials (8.4%; effect: 0.9%), *t*(37) = 1.320, *SE*_*diff*_ = .7, *p* = .195, $\eta_{p}^{2}=.04$. Thus, the switch cost was not statistically significant in errors with the current controls. Finally, *alt-AR* trials (13.7%) produced significantly *more* errors than *alt-AA* trials (effect: −5.2%), *t*(37) = −3.965, *SE*_*diff*_ = 1.3, *p* < .001, $\eta_{p}^{2}=.30$. This is consistent with the numerical pattern observed in the RT data, and suggests that there is a *cost* for repeating a physical key press if the conceptual response changes. This again suggests that the difference between *rep-AR* and *rep-AA* trials is not merely due to a physical repetition of the same key press, but instead due to the repetition of the same conceptual response.

### Discussion

The results of Experiment 1 demonstrate several interesting things. First, it is interesting that we observed robust switch costs with univalent stimuli. In some reports, this has not been observed [11, 42] (but see [43]), though our design did involve very different tasks (e.g., Jersild used univalent responses; see [33]). Secondly, we observed clear effects of stimulus and response repetitions that had notable effects on the switch cost. This is consistent with past reports [17, 29, 33], though our analysis broke down the data into finer comparisons than previously reported. Notice, critically, how these biases can lead to a systematic overestimation of the true effect of task switching. For instance, note that *rep-AA* trials were the slowest and most error prone task repetitions, whereas *alt-AA* trials were the fastest and least error prone task alternations. The switch cost is thus substantially reduced when considering this de-confounded (or less confounded) measure of task set switching. Stimulus and response repetitions thus unambiguously contribute to an increase in the switch cost. These feature repetition biases did not account for the entire switch cost, however. The remaining switch cost was still significant in the RT data, albeit much reduced. It was statistically eliminated in the errors, but numerically in the correct direction. As we will discuss shortly, however, there are still further considerations for this remaining switch cost that still leave open the possibility that switch costs are exclusively driven by low-level transitions and not higher-order control.

## Experiment 2

In Experiment 1, we were able to account for potential biases from stimulus repetitions, conceptual response repetitions, and physical response repetitions. Though switching between two stimulus sets has been done in some reports (e.g., [17]), one concern is that most task switching paradigms do not use this approach. Though we were able to rule out congruency effects by design, Experiment 1 is less comparable to the typical task switching experiment. The alternating runs design also has the potential limitation of possible “restart” costs occurring for the first trial in a fixed sequence of trials of the same task [44], which is not quite the same as the switch cost that is typically of interest. Indeed, we observed a relatively large switch cost with the classically-computed simple contrast between task alternations and task repetitions relative to other task switch experiments with distinct stimulus sets. This may have been due to the alternating runs design.

More critically for the current report, still other learning and memory biases might have been present that we did not assess. For instance, a task alternation entailed a change of stimulus type (i.e., from a letter to a digit, or vice versa), whereas a task repetition did not. It could be that a change in stimulus type entails a cost. Experiment 2 can partially control for this potential bias by making use of the same stimuli for two different decisions, as is more typical in the task switch literature. More specifically, digits were always the targets, and participants had to make either a parity or greater/less than five decision on each trial. For this, we abandoned the alternating runs design and used the cued version of the task switch paradigm, instead. That is, the colour of a rectangle presented before the target digit indicated the upcoming task. Thus, the same stimuli and physical key press responses can be used for both tasks.

The cued version of the task switch paradigm introduces another complication, however. It is known that the switch cost in this paradigm is largely, but not completely, driven by trials on which the cue on the previous trial matches the cue on the current trial [20, 45–48]. More concretely, responses are considerably faster when the cue on the previous trial matches the cue on the following trial, and cue repetitions can only occur when the task repeats. Thus, cue repetition benefits, another sort of feature integration effect, are directly confounded with the measure of task switching. Following previous reports, we use two cues for each task, which means that it is nevertheless possible for the task to repeat even when the cue changes (for some caveats with this approach, see [49]). We deliberately used non-meaningful cues (viz., coloured rectangles), because it is known that such cues produce much larger “true” switch costs than meaningful cues [50–52]. This thereby gives us the greatest possible chance of observing true effects of task set switching.

We again use the same (or similar) naming scheme for the conditions in this study, with the following alterations. The condition prefixes are “*cue*,” “*rep*,” and “*alt*,” referring, respectively, to the cases where both the task and cue repeat, just the task repeats (i.e., but not the cue), and when the task (and therefore cue) alternates. These three categories break further down into ten unique conditions when considering biases from stimulus and response repetitions. Again, “*A*” refers to an alternation, “*R*” to a repetition, and the two letters in the suffix (e.g., “*AR*”) refer to the stimulus and response, respectively.

The ten conditions that our manipulation produces are presented in Table 2. For task alternations, a full factorial combination of stimulus repetitions (repeat vs. alternation) and response repetitions (repeat vs. alternation) are possible, giving the conditions *alt-RR* (e.g., 1 parity → 1 magnitude, assuming that odd and less than five are mapped to the same key), *alt-AR* (e.g., 1 parity → 2 magnitude), *alt-RA* (e.g., 2 parity → 2 magnitude), and *alt-AA* (e.g., 4 parity → 2 magnitude). For task repetitions with or without a repeated cue, it is impossible to have the stimulus repeat without having the response repeat (though the reverse is possible), so the “*RS*” conditions were not observable (however, this could be possible in “transition cue” designs [53]; but see [54] for potential problems with the use of transition cues). This leaves *rep-RR* (e.g., 2 parity → 2 parity), *rep-AR* (e.g., 4 parity → 2 parity), and *rep-AA* (e.g., 3 parity → 4 parity) for task repetitions with no cue repetitions; and *cue-RR*, *cue-AR*, and *cue-AA* for task repetitions with cue repetitions (note that the same example trials apply to *cue* and *rep* trials, except that the colour of the cue also repeats in the former).

**Table 2:** Ten trial types in Experiment 2.

	Repetition Type				
Condition	Task	Cue	Stimulus	Conceptual Response	Physical Response
cue-RR	✓	✓	✓	✓	✓
cue-AR	✓	✓	✗	✓	✓
cue-AA	✓	✓	✗	✗	✗
rep-RR	✓	✗	✓	✓	✓
rep-AR	✓	✗	✗	✓	✓
rep-AA	✓	✗	✗	✗	✗
alt-RR	✗	✗	✓	✗	✓
alt-RA	✗	✗	✓	✗	✗
alt-AR	✗	✗	✗	✗	✓
alt-AA	✗	✗	✗	✗	✗

The number of potentially interesting comparisons that could theoretically be conducted between these ten conditions is quite large. We decided to restrict our data analyses to a few comparisons that we found of particular interest to reduce the potential of false positives, which would be inevitable when comparing all conditions in an indiscriminate manner. Some readers might think that one manner of analyzing the data would be to code each type of repetition as a separate binary regressor (i.e., one for task repetition vs. alternation, another for stimulus repetition vs. alternation, etc.), and then run a regression to independently measure the influence of each. Such an approach has been used to assess feature integration biases in the congruency sequence effect literature [55]. However, Schmidt and colleagues [6] have recently demonstrated an inherent problem with the statistical assumptions of such an approach. In particular, this approach ignores the potential interactions between different types of repetitions (e.g., a response repetition combined with a stimulus repetition). As we will see, considering these types of interactions is critically important for the current data. For instance, feature integration work [5] suggests that responding is faster on *complete repetitions*, where both the stimulus and response repeat, and on *complete alternations*, where both the stimulus and response change, relative to *partial repetitions*, where only the stimulus *or* the response repeats. Thus, the benefit for stimulus and response repetitions should not be additive.

We also consider congruency biases in a supplementary analysis. Of particular interest, we investigate whether the “pure” switch cost is modulated by stimulus congruency. Some reports have suggested that the switch cost is smaller for congruent trials (e.g., [33]), but this interaction seems to be fickle (for a discussion, see [56]) and was never tested with an “unbiased” measure of the switch cost.

### Methods

#### Participants

Twenty-six Ghent University undergraduates participated in the study in exchange for €8. None had participated in Experiment 1. This study was approved by the Ethics Committee of Ghent University. Participants provided written consent before participating. Note that a smaller sample size was collected given the greater number of observations per participants. Note, however, that this experiment is actually a replication of another experiment with more participants. The older experiment was exactly identical, except that there were only 200 trials per participant. Following the advice of two anonymous reviewers of an earlier version of this manuscript, the older experiment was dropped as reliability was too low. Generally, the older experiment produced exactly the same pattern of results as the current Experiment 2, save that some comparisons were not statistically significant in the older experiment. The most notable difference was that the “pure” task switch measure was not significant, albeit trending in the same direction. Readers interested in the results of this discarded experiment can contact the lead author for more information.

#### Apparatus

The apparatus was identical to Experiment 1.

#### Design

The materials and design were identical to Experiment 1 with the following exceptions. There were eight stimuli in total, consisting of the digits 1, 2, 3, 4, 6, 7, 8, and 9. In the parity judgment task, half of the participants responded to even digits with the “F” key and odd digits with the “J” key. The other half of the participants had the reverse mapping. In the magnitude task, all participants responded with the “F” key to digits less than five and with the “J” key for digits greater than five (i.e., in order to maintain S-R compatibility). Four task cues were used, consisting of a rectangle with a 5 pixel wide coloured border 40 x 30 pixels in dimension. The four colours were blue (0,0,255), yellow (255,255,0), orange (255,165,0), and pink (255,192,203). Which two colours were mapped to which task was randomized for each participant. As previously mentioned, these manipulations allow for ten types of trials: *cue-RR*, *cue-AR*, *cue-AA*, *rep-RR*, *rep-AR*, *rep-AA*, *alt-AA*, *alt-RA*, *alt-AR*, and *alt-AA*. Given the greater difficulty of the task, participants were also given a paper with a reminder of the mappings of conceptual responses to keys, and of colours to tasks.

#### Procedure

On each trial, participants saw a blank screen for 500ms, followed by the cue for 200ms, followed by the stimulus in the middle of the coloured rectangle for 3000ms or until a response was made (i.e., the cue remained on the screen). Participant were given 3000ms rather than 2000ms due to the greater difficulty of the task in Experiment 2. Following correct responses, the next trial immediately began. Following incorrect responses and trials in which participants failed to respond in 3000ms, “XXX” in white was presented for 500ms before the next trial. There were 800 trials in total, presented in four blocks of 200. Each block was separated by a five second pause. In contrast to Experiment 1, the trial sequence was random rather than a fixed alternating runs structure. Each trial was selected at random with replacement out of all the potential stimuli. Thus, there were approximately 50% alternations and 50% repetitions. Monsell and Mizon [25] highlighted the important role of transition frequency on the magnitude of switch costs. We would tend to agree with Logan and colleagues [47] that a random (50%) probability is ideal, as it does not introduce biased contingencies between the probability of one cue following another. As with other paradigms, such as the proportion congruent and congruency sequence tasks [9, 57–59], non-chance contingencies can introduce unintended learning confounds.

### Results

Mean correct RT and percentage error data were analyzed. All trials in which participants failed to respond in 3000 ms (2.6% of trials) and trials for which an error was made on the previous trial were excluded from analyses. One participant had below chance (48%) accuracy in the task. This participant was excluded from analyses, though this participant did not affect any of the important results reported below.

#### Response times

First, we conducted the traditional analysis, ignoring the distinctions between the repetition or alternation of cues, stimuli, or responses. There was a significant benefit for task repetitions (707ms) over task alternations (906ms; effect: 199ms), *t*(24) = 11.767, *SE*_*diff*_ = 17, *p* < .001, $\eta_{p}^{2}=.85$. Thus, we replicated the standard switch cost.

Next, we consider the influence of cue repetitions on the switch cost by dividing the task repetitions into trials where the cue did or did not repeat. A three condition (*cue*, *rep*, *alt*) ANOVA was significant, *F*(2,48) = 116.451, *MSE* = 4017, *p* < .001, $\eta_{p}^{2}=.83$. Again, we observed that task repetitions without cue repetitions (785ms) were significantly faster than task alternations (906ms; effect: 121ms), *t*(24) = 8.474, *SE*_*diff*_ = 14, *p* < .001, $\eta_{p}^{2}=.75$. Thus, we observed a task switch cost independent of cue repetition benefits, but it was greatly reduced in magnitude. We also observed that task repetitions *with* cue repetitions (633ms) were considerably faster than task repetitions without cue repetitions (effect: 152ms), *t*(24) = 9.251, *SE*_*diff*_ = 17, *p* < .001, $\eta_{p}^{2}=.78$, demonstrating that over half of the switch cost in this paradigm is driven by cue repetition benefits.

**Fig 4:**
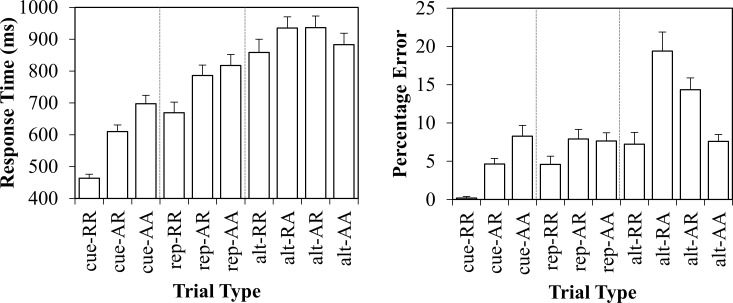
Experiment 2 responses times in milliseconds (left) and percentage errors (right) with standard error bars.

Next, we consider the more fine-grained analyses. These data are presented in the left panel of Fig 4. The ten unique trial types were submitted to an ANOVA. The main effect of condition was significant, *F*(9,216) = 91.193, *MSE* = 6476, *p* < .001, $\eta_{p}^{2}=.79$.

For the finer comparisons, first we consider trials in which the task alternated. The four unique trial types represent a factorial combination of stimulus repetitions (repetition vs. alternation) and response repetitions (repetition vs. alternation). Inserting these two factors into an ANOVA did not produce a significant main effect of stimulus repetition, *F*(1,24) = .738, *MSE* = 4669, *p* = .399, $\eta_{p}^{2}=.03$, or of response repetition, *F*(1,24) = .823, *MSE* = 4891, *p* = .373, $\eta_{p}^{2}=.03$. However, there was a significant interaction between the two, *F*(1,24) = 20.466, *MSE* = 5213, *p* < .001, $\eta_{p}^{2}=.46$. This interaction was due to faster responses to trials in which the same response was made to the same stimulus (complete repetitions) and trials in which a different response was made to a different stimulus (complete alternations). Specifically, *alt-RR* trials were significantly faster (858ms) than *alt-RA* trials (935ms; effect: 77ms), *t*(24) = 2.933, *SE*_*diff*_ = 26, *p* = .007, $\eta_{p}^{2}=.26$, and *alt-AR* trials (936ms; effect: 78ms), *t*(24) = 3.845, *SE*_*diff*_ = 20, *p* < .001, $\eta_{p}^{2}=.38$. Additionally, *alt-AA* trials were significantly faster (883ms) than *alt-AR* trials (effect: 53ms), *t*(24) = 5.359, *SE*_*diff*_ = 10, *p* < .001, $\eta_{p}^{2}=.54$, and *alt-RA* trials (effect: 52ms), *t*(24) = 2.643, *SE*_*diff*_ = 20, *p* = .014, $\eta_{p}^{2}=.23$. Thus, the overall pattern matches that of the feature integration account.

Next we consider trials in which the task repeats with a cue repetition (*cue* condition) or without (*rep* condition). We performed a three condition (*RR*, *AR*, *AA*) by two cue repetition (*cue* vs. *rep*) ANOVA. This produced a very large and significant cue repetition effect, *F*(1,24) = 87.874, *MSE* = 11916, *p* < .001, $\eta_{p}^{2}=.79$. The main effect of condition was also significant, *F*(2,48) = 133.504, *MSE* = 3575, *p* < .001, $\eta_{p}^{2}=.85$, as was the interaction, *F*(2,48) = 10.919, *MSE* = 2148, *p* < .001, $\eta_{p}^{2}=.31$. Decomposing this interaction, there was a significant response repetition benefit (i.e., *SS* vs. *SR*), *F*(1,24) = 38.621, *MSE* = 2275, *p* < .001, $\eta_{p}^{2}=.62$, which was significantly larger in the *cue* condition than in the *rep* condition, *F*(1,24) = 14.655, *MSE* = 1357, *p* < .001, $\eta_{p}^{2}=.38$. There was also a significant stimulus repetition benefit (i.e., *SR* vs. *RR*), *F*(1,24) = 166.623, *MSE* = 2599, *p* < .001, $\eta_{p}^{2}=.87$, but this did not significantly differ in the *cue* and *rep* conditions, *F*(1,24) = 2.477, *MSE* = 2083, *p* = .129, $\eta_{p}^{2}=.09$.

As a final analysis, we now consider a comparison between task alternations (*alt*) and task repetitions without cue repetitions (*rep*). Specifically, we perform a task switch (*alt* vs. *rep*) by condition (*RR*, *AR*, *AA*) ANOVA. This analysis produced a significant main effect of condition, *F*(2,48) = 44.518, *MSE* = 3204, *p* < .001, $\eta_{p}^{2}=.65$, which has already been broken down in the analyses above. Most importantly, this ANOVA also revealed a significant task switch cost, *F*(1,24) = 67.080, *MSE* = 10029, *p* < .001, $\eta_{p}^{2}=.74$. The interaction was also significant, *F*(2,48) = 13.810, *MSE* = 3629, *p* < .001, $\eta_{p}^{2}=.37$. Decomposing this interaction, we are particularly interested to know whether there is a switch cost on *AA* trials, where neither the conceptual nor physical response repeats. This critical comparison was significant (*rep-AA*: 817ms; *alt-AA*: 883ms; effect: 66ms), *t*(24) = 4.469, *SE*_*diff*_ = 15, *p* < .001, $\eta_{p}^{2}=.45$. Thus, a “pure” switch cost was observed. It is also noteworthy that this measure of “true” switch costs is much smaller than that observed when just controlling for cue repetition benefits (121ms). Thus, like the preceding experiment, it is clear that elimination of stimulus and response repetitions greatly attenuates the switch cost, even after ruling out cue repetition benefits. There were also significant differences between *rep-AR* (786ms) and *alt-AR* (936ms; effect: 150ms), *t*(24) = 7.876, *SE*_*diff*_ = 19, *p* < .001, $\eta_{p}^{2}=.72$, and between *rep-RR* (669ms) and *alt-RR* (858ms; effect: 189ms), *t*(24) = 6.670, *SE*_*diff*_ = 28, *p* < .001, $\eta_{p}^{2}=.65$. Note, however, that these last two contrasts do not control for conceptual response repetitions (i.e., participants make the same conceptual response with the same key in the *rep* condition, and a different conceptual response with the same key in the *alt* condition).

As an additional consideration, congruency can be coded as another factor. While *alt-RA* trials are inherently incongruent (viz., as they entail responding to the same stimulus with a different response) and *alt-RR* trials are inherently congruent (viz., because they require responding to the same stimulus with the same response in both tasks), the remaining eight trial types can be congruent or incongruent. Table 3 presents the full dataset. Here, however, our primary concern is whether the “pure” switch cost that we observed between *alt-AA* and *rep-AA* trials is modified by congruency. A two switch condition (*alt* vs. *rep*) by two congruency (congruent vs. incongruent) ANOVA produced a significant main effect of congruency, *F*(1,24) = 37.690, *MSE* = 3918, *p* < .001, $\eta_{p}^{2}=.61$, indicating overall faster responses to congruent relative to incongruent trials. Interestingly, the ANOVA did not produce a significant interaction, *F*(1,24) = .003, *MSE* = 2922, *p* = .960, $\eta_{p}^{2}<.01$. Furthermore, the switch cost was significant for both congruent trials, *t*(24) = 3.771, *SE*_*diff*_ = 19, *p* < .001, $\eta_{p}^{2}=.37$, and incongruent trials, *t*(24) = 4.085, *SE*_*diff*_ = 17, *p* < .001, $\eta_{p}^{2}=.41$.

**Table 3:** Congruent and incongruent trials in Experiment 2.

	Response Time				Errors			
Condition	Congruent		Incongruent		Congruent		Incongruent	
	*Mean*	*SE*	*Mean*	*SE*	*Mean*	*SE*	*Mean*	*SE*
cue-RR	458	15	470	13	0.0	0.0	0.4	0.4
cue-AR	588	22	631	22	1.6	0.8	7.4	1.1
cue-AA	673	25	724	30	6.1	1.2	10.7	1.8
rep-RR	660	33	693	43	4.2	1.4	5.2	1.6
rep-AR	780	37	791	33	4.2	1.0	11.2	1.7
rep-AA	781	33	859	39	5.0	0.9	10.3	1.5
alt-RR	858	42			7.2	1.5		
alt-RA			935	35			19.4	2.5
alt-AR	905	39	964	37	6.6	0.9	20.0	2.3
alt-AA	852	37	928	36	3.9	0.7	12.2	1.4

#### Percentage error

First, we conducted the traditional analysis, ignoring the distinctions between the repetition or alternation of cues, stimuli, or responses. There was a significant benefit for task repetitions (6.7%) over task alternations (11.1%; effect: 4.4%), *t*(24) = 8.935, *SE*_*diff*_ = 0.5, *p* < .001, $\eta_{p}^{2}=.77$. Thus, we replicated the standard switch cost in errors.

Next, we consider the influence of cue repetitions on the switch cost by dividing the task repetitions into trials where the cue did or did not repeat. A three condition (*cue*, *rep*, *alt*) ANOVA was significant, *F*(2,48) = 38.836, *MSE* = 5.5, *p* < .001, $\eta_{p}^{2}=.62$. Consistent with the response times, we observed that task repetitions without cue repetitions (8.0%) generated less errors than task alternations (11.1%; effect: 3.1%), *t*(24) = 5.350, *SE*_*diff*_ = 0.6, *p* < .001, $\eta_{p}^{2}=.54$. Again, this indicates a task switch cost independent of cue repetition benefits. Task repetitions *with* cue repetitions (5.3%) also generated significantly less errors than those without (effect: 2.7%), *t*(24) = 3.553, *SE*_*diff*_ = 0.8, *p* = .002, $\eta_{p}^{2}=.34$.

Similar to the response times, the ten unique trial types were submitted to an ANOVA. The data are presented in the right panel of Fig 4. The main effect of condition was significant, *F*(9,216) = 23.017, *MSE* = 30.7, *p* < .001, $\eta_{p}^{2}=.49$.

Again, we first consider trials in which the task (and cue) alternated. The stimulus repetition (repetition vs. alternation) by response repetition (repetition vs. alternation) ANOVA produced only a marginal stimulus repetition effect, *F*(1,24) = 3.003, *MSE* = 61, *p* = .096, $\eta_{p}^{2}=.11$, and a marginal response repetition effect, *F*(1,24) = 3.488, *MSE* = 39, *p* = .074, $\eta_{p}^{2}=.13$. Critically, the interaction was significant, *F*(1,24) = 33.953, *MSE* = 66, *p* < .001, $\eta_{p}^{2}=.59$. This interaction was driven by reduced errors when the same response was made to the same stimulus (complete repetitions) and when a different response was made to a different stimulus (complete alternations). Specifically, *alt-RR* trials generated significantly less errors (7.2%) than *alt-RA* trials (19.4%; effect: 12.2%), *t*(24) = 4.149, *SE*_*diff*_ = 2.9, *p* < .001, $\eta_{p}^{2}=.42$, and *alt-AR* (14.4%; effect: 7.2%), *t*(24) = 4.420, *SE*_*diff*_ = 3.8, *p* < .001, $\eta_{p}^{2}=.45$. Additionally, *alt-AA* generated significantly less errors (7.6%) than *alt-AR* (effect: 6.8%), *t*(24) = 5.452, *SE*_*diff*_ = 1.2, *p* < .001, $\eta_{p}^{2}=.55$, and *alt-RA* (effect: 11.8%), *t*(24) = 4.893, *SE*_*diff*_ = 2.4, *p* < .001, $\eta_{p}^{2}=.50$. Thus, the errors replicate the pattern in the response times.

Next we consider trials in which the task repeats with a three condition (*RR*, *AR*, *AA*) by two cue condition (*cue* vs. *rep*) ANOVA. Again, we observed a significant cue repetition effect, *F*(1,24) = 20.986, *MSE* = 10, *p* < .001, $\eta_{p}^{2}=.47$, and a significant main effect of condition, *F*(2,48) = 23.537, *MSE* = 17, *p* < .001, $\eta_{p}^{2}=.50$. The interaction was also significant, *F*(2,48) = 6.553, *MSE* = 13, *p* = .003, $\eta_{p}^{2}=.21$. Decomposing this interaction, there was a significant response repetition benefit (i.e., *AA* vs. *AR*), *F*(1,24) = 5.310, *MSE* = 13, *p* = .030, $\eta_{p}^{2}=.18$, which was significantly larger in the *cue* condition than in the *rep* condition, *F*(1,24) = 6.912, *MSE* = 14, *p* = .015, $\eta_{p}^{2}=.22$. There was also a significant stimulus repetition benefit (i.e., *AR* vs. *RR*), *F*(1,24) = 28.347, *MSE* = 13, *p* < .001, $\eta_{p}^{2}=.54$, but this did not significantly differ in the *cue* and *rep* conditions, *F*(1,24) = 1.056, *MSE* = 7, *p* = .314, $\eta_{p}^{2}=.04$.

Again, we finally consider a comparison between task alternations and task repetitions without cue repetitions in a task switch (*alt* vs. *rep*) by condition (*RR*, *AR*, *AA*) ANOVA. This analysis produced a significant main effect of condition, *F*(2,48) = 13.295, *MSE* = 27, *p* < .001, $\eta_{p}^{2}=.36$, which has already been broken down in the analyses above. Most importantly, this ANOVA also revealed a significant task switch cost, *F*(1,24) = 18.060, *MSE* = 19, *p* < .001, $\eta_{p}^{2}=.43$. The interaction was also significant, *F*(2,48) = 6.826, *MSE* = 19, *p* = .002, $\eta_{p}^{2}=.22$. Decomposing this interaction, we are particularly interested to know whether there is a task switch cost on *AA* trials, where neither the conceptual or physical response repeats. This critical comparison was not significant (*rep-AA*: 7.6%; *alt-AA*: 7.6%; effect: <0.1%), *t*(24) = .054, *SE*_*diff*_ = 0.7, *p* = .957, $\eta_{p}^{2}<.01$. Thus, no “pure” switch cost was observed, in contrast to the response times. This again demonstrates that elimination of stimulus and response repetitions greatly attenuates the switch cost, even after ruling out cue repetition benefits. There was a significant difference between *rep-AR* (7.9%) and *alt-AR* (14.4%; effect: 6.5%), *t*(24) = 6.606, *SE*_*diff*_ = 1.0, *p* < .001, $\eta_{p}^{2}=.65$, but not between *rep-RR* (4.6%) and *alt-RR* (7.2%; effect: 2.6%), *t*(24) = 1.496, *SE*_*diff*_ = 1.8, *p* = .148, $\eta_{p}^{2}=.09$. Note again that these last two contrasts do not control for conceptual response repetitions.

Given the lack of a switch cost, congruency was not analysed further. However, the full error dataset is presented in Table 3.

### Discussion

The results of Experiment 2 provide further support for the notion that feature repetitions play a sizable role in the switch cost. First, we replicated the finding of Logan and Bundesen [19] and Mayr and Kliegl [48] that the cued switch cost is sizably reduced when removing the influence of cue repetitions (i.e., from 199ms to 121ms in response times, and 4.4% to 3.1% in errors). Second, we also replicated the finding that a relatively large switch cost remains after controlling for cue repetitions when non-meaningful cues are used [50–52]. Third, we observed that the effect is further reduced when eliminating stimulus, physical response, and conceptual response repetitions (66ms in response times and <0.1% in errors). What these results clearly show is that the vast majority of the switch cost can be explained by feature integration biases. This was further underscored by analyses of complete repetitions, complete alternations, and partial repetitions, which revealed evidence for a benefit for the first two relative to the latter (see also, [29]). Like Experiment 1, our results again also indicated the importance of the distinction between a conceptual response repetition (e.g., even→even) and a simple response key repetition [36]. Additionally, we found evidence that response repetitions produced larger effects on cue repetition trials than on task repetitions without cue repetitions, consistent with previous results [36]. Most critically, a “pure” switch cost *was* observed in Experiment 2, albeit of a reduced magnitude and only in the response times. Collectively, the results demonstrate that the majority of the switch cost is explainable by cue, stimulus, and response repetitions. However, a switch cost is still present. Congruency was not found to impact the switch cost, in contrast to some prior reports (e.g., [33]). Notably, the present report investigated the effect of congruency on an unbiased measure of the switch cost, which may explain the discrepancy.

## General Discussion

The goal of this study was to investigate for the first time the combined impact of various types of feature integration biases (e.g., cue repetition, stimulus-response bindings, etc.) on the switch cost. Our novel integrative approach highlights the important point that one can only understand the true extent to which feature integration plays a role in the switch cost by controlling for all feature integration effects simultaneously. In two experiments, we observed very large impacts of feature integration biases on the switch cost. In Experiment 1, we observed that performance is substantially increased in task repetitions when both the stimulus and response repeat from the previous trial. When only the response (but not the stimulus) repeats, this leads to a benefit for a task repetition, but a cost for a task alternation. Thus, these stimulus and response repetition biases work to systematically increase the switch cost. We also found evidence that conceptual response repetitions (e.g., even→even) have effects above and beyond those for a simple response key repetition. Importantly, there was still a task switch cost for the trials in which neither the stimulus nor the response repeated. However, this effect was considerably reduced. It also remains possible that some or all of the remaining effect may have been due to biases from alternating stimulus types on a task alternation (e.g., a digit followed by a letter, or vice versa) versus repeating the same stimulus types on a task repetition.

Experiment 2 was able to get around the stimulus set problem by using a cued version of the task switch paradigm. The same stimuli were responded to with different tasks. In this experiment, we again found clear evidence of interactive stimulus and response biases. For instance, *complete repetitions* (both the stimulus and response repeat) and *complete alternations* (both change) benefited relative to *partial repetitions* (either the stimulus or the response repeats; see also, [29]). We also replicated the finding of sizeable cue repetition effects [19, 20] and the finding of response repetition benefits with a conceptual response repetition (i.e., on a task repetition) and response repetition costs with a conceptual response alternation (i.e., on a task alternation) [36]. Most critically, we controlled for all of these stimulus, response, and cue repetitions effects simultaneously for the first time in the present report and we found that the remaining task switch cost was much reduced. Indeed, it was only significant in the response times of Experiment 2. Taken together, our results demonstrated that considering the feature integration biases of stimuli, physical responses, conceptual responses, and cues explain the majority, but not all, of the switch cost. Whether appeals to higher-order cognitive control mechanisms are necessary to explain the remaining switch cost is uncertain (as will be discussed shortly). However, our integrative approach clearly indicates that lower-level integration biases explain most of the variance in the switch cost. More importantly, the novel suggestion of our work is that controlling for feature integration biases should be standard practice in task switching experiments when one is interested in cognitive processes other than feature integration.

### An Integrative Account

We suggest that these results fit nicely with an episodic learning (e.g., [60]) or event file [5] account of task switching. To the extent that previous memory bindings, particularly from immediately preceding trials, overlap with events on the current trial, performance can be aided or impaired. As we have shown, these biases happen to work more in the favour of task repetitions and more to the detriment of task alternations. Specifically, stimulus repetitions *always* require a response repetition (i.e., “complete repetition”) on a task repetition, but often require a different response (i.e., “partial repetition”) on a task alternation. Similarly, physical key repetitions *always* entail a conceptual response repetition on a task repetition, but *always* entail a conceptual response alternation on a task alternation. Finally, cue repetitions are only possible on a task repetition. Thus, nearly all feature integration biases work to the advantage of task repetitions and to the disadvantage of task alternations.

It is important to note that stimulus, response, and cue repetitions do not necessarily represent “confounds” or “bias” in the switch cost, depending on the goal of the researcher. The switch cost is obviously a real and interesting effect, regardless of what mechanism drives it. Indeed, feature integration effects are interesting in their own right. If feature integration biases such as these explain the entire switch cost, then the switch cost is no less real. Feature integration “biases” are merely one potential account of origin of the switch cost. However, our results do suggest some complications for the case in which the goal of the research *is* to specifically study the effect of switching the task itself, as would be implied by several important theories of the switch cost. For instance, the idea that the switch cost comes about via a reconfiguration of the task set with a switch [17] or via proactive interference [18] should imply that a switch cost is still observable after removing all trials with a stimulus, response, or cue repetition from the data. We did observe this. However, performing a task switch experiment without the sort of controls used in the current experiments is simply insufficient to draw any inferences about higher-order control.

For instance, consider what happens when a researcher uses the standard (biased) design, manipulates Variable X, and observes that it impacts the size of the switch cost. The researcher might want to conclude that Variable X influences cognitive control. However, this is not a warranted conclusion, because Variable X may actually be influencing the binding processes responsible for feature integration effects, instead. Indeed, given that more of the switch cost is explained by feature integration effects than anything else, it may actually be *more* likely that Variable X impacts feature integration. As an example, the finding that switch costs decay with increasing response-cue intervals (RCI) might indicate, on the one hand, that task set reconfiguration takes time [61]. On the other hand, the decay with RCI might indicate decreases in episodic binding with changes in the RCI [62]. Similarly, the finding that switch costs are smaller with compatible stimulus-response mappings (e.g., auditory-vocal and visual-manual) than with incompatible stimulus-response mappings (e.g., auditory-manual and visual-vocal) [63, 64] might be (very slightly) reinterpreted as indicating that two sets of bindings (one for each task) are easier to keep separate with two distinct compatible input-output relations, but the bindings from the two tasks get crossed with incompatible mappings. While not all task switching experiments are as heavily biased as those used in the present investigation [26–28], it is common practice to use measures of task set switching that contain at least some of these biases. We suggest that this should change in all future research on the topic, as has gradually become the case in the congruency sequence effect literature (for a review, see [10]).

Unfortunately, the types of experiments used in the current report are not really ideal for this sort of research, as the data must be split multiple ways to rule out all “confounds.” Statistical power therefore becomes a concern. This is particularly the case in the cued version of the task, where data must be split more ways. Though more trials could of course be run to increase the number of trials that “survive” the trimming, it is still problematic how small a proportion of trials can be used to assess the “pure” switch cost (34.375% for the critical comparisons in Experiment 2, before taking into account errors), particularly when one is interested in orthogonally manipulating other factors, such as the cue-stimulus interval or response-cue interval (i.e., because the data must be divided even further).

One possible solution to this predicament might be to take inspiration from recent work on congruency sequence effects. The *congruency sequence effect* (i.e., a reduced congruency effect following an incongruent trial [65]) is also plagued with feature repetition biases [7–9]. One recent approach to dealing with this issue is to split up the task into two alternating stimulus and response sets [66–71], such that it is *impossible* to have repetitions of stimuli or responses. In the case of task switching, this could be (partially) achieved by splitting the stimulus set in half (e.g., 1469 and 2378) and alternating between these two sets on even and odd trials. Task alternations and repetitions can still occur orthogonally to this, but the stimuli can never repeat (alternatively, a large stimulus set can be used to prevent repetitions of stimuli [25, 56]). Similarly, in the cued version of the task, two cues (one for each task) could occur only on odd trials and the other two only on even trials. Thus, the cue could never repeat. Response repetitions, however, are more inherently “confounded” in task switching paradigms. These response repetitions cannot be ignored, particularly given the large systematic biases from conceptual response repetitions observed in the current manuscript. Thus, some trimming would still be necessary. Still, such manipulations would greatly reduce the need to delete large portions of data when assessing a “pure” switch cost.

### Congruency

With the modified design mentioned above, attention to the impact of *congruency* on the task switch effect is probably warranted [16, 17, 33]. We observed clear congruency effects, but did not find evidence of an interaction between congruency and switching in our data. This might be because congruency was completely orthogonal to the task switch factor in our “unbiased” measure of the switch cost, and was also measured in the absence of any feature integration biases. This is unlike past reports. That said, consideration of congruency biases in addition to feature integration biases in future research is desirable.

One approach to modelling congruency biases is to assume that a stimulus both (a) strongly biases the task-relevant response, and (b) weakly biases the task-irrelevant response [41] (see also, [23, 72]). This leads to an advantage for congruent trials, where both tasks point to the same (key) response, relative to incongruent trials, where both response keys are biased. In fact, this notion is highly interrelated with the learning perspective suggested here. A stimulus is only “congruent” to the extent that it is consistently bound with the same response, and “incongruent” to the extent that it is not. In this sense, congruency is episodic binding beyond the immediately preceding trial.

### Remaining Switch Costs

Of course, we still observed a remaining switch cost in our response time data. Although this is certainly consistent with the notion that higher-order control processes may play some role in the switch cost, it is possible that this remaining switch cost is also due to feature integration effects. For instance, Schneider and Logan [23] suggested that when two arbitrary cues are assigned to each task, the cues of the same task might prime the same mediator (for related work on inter-cue priming, see [22]). For instance, blue and yellow might prime a “parity” mediator, and retrieval of the mediator can be easier when the task repeats (i.e., mediator repetition) relative to when the task alternates (i.e., mediator alternation). It is possible that mediator priming might explain the remaining switch cost we observed in Experiment 2, though it is difficult to distinguish between such mediator priming and control via task sets. Future research might aim to distinguish between these two possibilities.

The above also relates to the priming and inhibition account [27, 34]. In addition to general inhibition of responses that were activated, this account explains response repetition effects by assuming that the *stimulus category* (e.g., “odd,” “less than five,” etc.) of one trial remains active on the following trial, and this category priming makes it easier to make the same category decision on the next trial (which is only ever the case on a task repetition). Of course, our “pure” measure of the switch cost specifically discards all trials in which the stimulus category repeats (i.e., because response repetitions are excluded), so it is unlikely that category priming confounded our measure. However, the notion that the stimulus category and/or the mediator (e.g., “parity” or “magnitude”) plays a role in the typical switch cost is generally consistent with our feature integration account.

Similarly, inter-cue priming might also occur. Each cue is linked in memory to the conceptual responses of just one task. For instance, if blue and yellow cues are consistently paired with “even” and “odd” conceptual responses, then these two cues might become linked via their overlap with conceptual responses. This could lead to inter-cue priming. Indeed, it has been observed that if Stimulus A is associated with Response X (i.e., Stimulus A ↔ Response X) and Stimulus B is also associated with Response X (i.e., Stimulus B ↔ Response X), then Stimulus A and Stimulus B become associated via an indirect association (e.g., Stimulus A ↔ Response X ↔ Stimulus B) [73]. Thus, cue alternations may be less “pure” on a task repetition than on a task alternation. Relatedly, because each cue is only associated with two conceptual responses (e.g., “even” and “odd”), both conceptual responses may be primed on cue presentation. Of course, only one conceptual response will be selected, but the other conceptual response might be partially primed as well [33, 41]. Such passive conceptual response priming might also contribute to the switch cost. These speculative ideas could be investigated in future task switching research.

As the above analysis indicates, it may or may not be necessary to appeal to the notion of task set reconfiguration costs [16, 17] or even to retrieval of previous task sets [38–40]. Indeed, it is possible that there are no active higher-order control processes that distinguish task alternations from task repetitions. Episodic retrieval biases from lower-level feature repetitions might provide a complete and highly parsimonious account of the switch cost. We suggest this hesitantly, however, as more work is still needed to investigate this possibility more closely. Indeed, the present results clearly indicated that a reliable switch cost does remain after controlling for cue, stimulus, and response repetitions, and this remaining effect might, in fact, be due to some form of task set control. It should be clear, however, that the “true” task switch cost that remains after controlling for lower-level feature biases is of a much smaller magnitude in comparison to results with the standard, biased approach of analysing data.

Of course, intermediate views also exist. For instance, some authors suggest that the task set might be encoded into the episodic representation of a trial [38–40]. For instance, Waszak and colleagues presented participants with incongruent picture-word stimuli, with alternating picture naming and word reading as the two tasks. The switch cost was particularly large for word reading when words had been previously presented as distracters in picture naming. They interpret their findings as indicating that the stimuli become linked to the previously-experienced task in memory, thus biasing the wrong task when a stimulus has previously been presented as a distracter in the other task. In this way, control and memory binding are intimately interrelated (see also, [74, 75]). A caveat with their experimental approach is that their effects could alternatively be interpreted in terms of stimulus-response bindings formed between the previous presentation of the word with the response that had been made to the picture [76]. Nevertheless, the notion that participants not only encode the cues, stimuli, and responses into episodes, but also encode task and/or control settings is one potential interpretation of both: (a) the large feature integration effects we observed, and (b) the remaining switch cost.

Similarly, it could be the case that some higher-order control processes do explain the remaining switch cost we observed, but these higher-order processes may be unrelated to task sets. For instance, participants might have a greater expectancy for a task repetition over a task alternation. This might then lead to a priming of the conceptual responses for the repeated task, producing a benefit for task repetitions over task alternations. For instance, when participants are allowed to pick which task they do on any given trial, they have a tendency to repeat rather than switch tasks [77, 78]. Whether the same expectancy biases would occur in the cued version of the paradigm is less clear, however. Indeed, the objective task switch frequency is 50%. That said, expectations for repetitions has been argued to occur under the same scenario in other tasks [65].

### Concluding Remarks

Mental flexibility is argued to be a key part of executive functioning [1–3]. However, studies of higher-order cognitive functions can often be entangled with unintended biases. In particular, lower-level learning processes often masquerade as higher-order control processes. We argue that it is critical to take into consideration the basic learning and memory biases that may confound analyses aimed to assess higher-order cognitive control processes in order to prevent false confidence that control is producing an effect. Indeed, our integrative approach demonstrates that it is important to consider all sources of feature integration biases concurrently in order to obtain a truly-unbiased measure of the task switch cost. This is not only true for task switching, but also for other paradigms that aim to assess cognitive control, such as the proportion congruency and congruency sequence effects [10]. Indeed, controls for feature integration biases have become standard practice in other literatures, and we argue for the first time that the same should be true in task switching research. If there are low-level regularities in a task, then they will almost certainly affect performance. If these biases systematically confound a key comparison, as they do with the switch cost, then it becomes difficult to know what one is truly studying. Since these low-level biases are very rarely what the researcher is actually interested in, rigorous controls, comparable to the ones used in the current manuscript, are necessary for every experiment aiming to study higher-order control. In the specific case of task switching, we demonstrated that these low-level biases explain most of the switch cost. That is, repetitions of cues, stimulus-response bindings, and conceptual-physical response bindings all serve to systematically benefit performance on a task repetition and to impair performance on a task alternation. These biases can be parsimoniously explained in a simple episodic storage and retrieval model of performance. Our data do not rule out the possibility that control processes explain at least part of the switch cost (i.e., particularly given that we found remaining switch costs), but we believe appeals to high-order control processes when explaining switch costs should be made cautiously. Occam’s razor should favour the simpler account.

## Supporting Information

S1 Data Experiment 1 raw E-Prime 2 data file (task switch.emrg2). (EMRG2)

S2 Data Experiment 2 raw E-Prime 2 data file (switch3.emrg2). (EMRG2)
